# RETRACTION: Suppression of lncRNA OIP5‐AS1 Attenuates Apoptosis and Inflammation, and Promotes Proliferation by Mediating miR‐25‐3p Expression in Lipopolysaccharide‐Induced Myocardial Injury

**DOI:** 10.1155/ancp/9805806

**Published:** 2026-06-10

**Authors:** Analytical Cellular Pathology

RETRACTION: J. Ma, H. Qian, and H. Zou, “Suppression of lncRNA OIP5‐AS1 Attenuates Apoptosis and Inflammation, and Promotes Proliferation by Mediating miR‐25‐3p Expression in Lipopolysaccharide‐Induced Myocardial Injury,” *Analytical Cellular Pathology*, 2023, 3154223, https://doi.org/10.1155/2023/3154223.

The above article, published online on 20 March 2023 in Wiley Online Library (wileyonlinelibrary.com), has been retracted by agreement between the Journal Editor‐in‐Chief, Dimitrios Karamichos; and John Wiley & Sons Ltd. The retraction has been agreed due to concerns that have been reported on PubPeer [1], specifically regarding Figure 2h in which there are unexpected, repeated regions between the DAPI and Merge LPS Blank panels, and the DAPI and Merge LPS + sh‐NC panels. The publisher has confirmed these concerns independently.

Additionally, concerns have been reported on PubPeer regarding the apoptosis measurements [1].

The authors did not provide a response to the concerns, and after assessment by the Editorial Board the results and conclusions cannot be considered reliable. The article is therefore retracted.

The authors did not respond to the retraction.

## References

[1] tanganyikensis I. , Suppression of lncRNA OIP5-AS1 Attenuates Apoptosis and Inflammation, and Promotes Proliferation by Mediating miR-25-3p Expression in Lipopolysaccharide-Induced Myocardial Injury, *PubPeer*, 2024, https://pubpeer.com/publications/505CFF56938BD58CEA83F8E7C6F8DE. 10.1155/2023/3154223 PMC10042636 36994450

